# Evidence for co-translational misincorporation of non-canonical amino acid hydroxyproline in recombinant antibodies produced in Chinese Hamster Ovary (CHO) cell lines

**DOI:** 10.1371/journal.pone.0241250

**Published:** 2020-10-29

**Authors:** Shanta Boddapati, Jason Gilmore, Kyle Boone, John Bushey, Jonathan Ross, Brian Gfeller, William McFee, Romesh Rao, Greg Corrigan, Aaron Chen, Howard Clarke, John Valliere-Douglass, Swapnil Bhargava

**Affiliations:** 1 Process Sciences, Seattle Genetics Inc, Bothell, WA, United States of America; 2 Upstream Manufacturing, Seattle Genetics Inc, Bothell, WA, United States of America; Aarhus University, DENMARK

## Abstract

With the advent of highly sensitive technologies such as tandem mass spectrometry and next-generation sequencing, recombinant antibodies are now routinely analyzed for the presence of low-level sequence variants including amino acid misincorporations. During mAb cell culture process development, we found that proline was replaced with the non-canonical amino acid, hydroxyproline, in the protein sequence. We investigated the relationship between proline content in the cell culture media and proline sequence variants and found that the proline concentration was inversely correlated with the amount of sequence variants detected in the protein sequence. Hydroxyproline incorporation has been previously reported in recombinant proteins produced in mammalian expression systems as a post-translational modification. Given the dependency on proline levels, the mechanism was then investigated. To address the possibility of co-translational misincorporation of hydroxyproline, we used tandem mass spectrometry to measure incorporation of stable-isotope labelled hydroxyproline added to the feed of a production bioreactor. We discovered co-translational misincorporation of labelled hydroxyproline in the recombinant antibody. These findings are significant, since they underscore the need to track non-canonical amino acid incorporation as a co-translational event in CHO cells. Understanding the mechanism of hydroxyproline incorporation is crucial in developing an appropriate control strategy during biologics production.

## Introduction

Chinese hamster ovary (CHO) cells are the main mammalian expression system for protein production used by the biopharmaceutical industry [1, 2]. A primary expectation from biotherapeutic manufacturing in this industry is producing product with consistent clinical performance. This is achieved by developing manufacturing processes with consistent product quality thereby ensuring the safety and efficacy of the drugs [3]. Drug product release specifications define an acceptable range of product quality attributes that can be tolerated to maintain product safety and efficacy. Thus, it is critical that the manufacturing process robustly delivers product that meets release acceptance criteria. In-depth product characterization is necessary to develop a rigorous understanding of product quality attributes. Significant advances have been made to recombinant protein analytical technology to enable the detection and quantification of micro-heterogeneity in product quality, for example low-level sequence variants (SV) [4, 5]. Sequence variants can arise from DNA mutations or protein mistranslation. Mistranslation occurs typically through misincorporation of non-cognate amino acids by codon mispairing or from mischarged tRNA (misacylation) [6, 7]. While DNA mutations result in amino acid variants at a single site, misincorporations are distributed across the translated protein. Multiple amino acids, such as asparagine and tyrosine to name a few, have been previously reported to be erroneously substituted by non-cognate amino acids in recombinant antibodies produced in CHO cells [8, 9]. Incorporation of non-proteinogenic amino acids into proteins produced in mammalian expression systems is less understood.

Protein translation in mammalian cells is typically a high-fidelity process. This process relies on accuracy in the esterification of a cognate amino acid to tRNA upon recognition by the appropriate aminoacyl-tRNA synthetase. In addition, multiple pre- and post-transfer proofreading steps can result in hydrolysis of a non-cognate amino acid, thus reducing the probability of translation errors [10, 11]. Amino acid misincorporations can be exacerbated based on the specific amino acid and the context of the cellular environment influencing protein translation. Misacylation propensity also differs among different species depending on efficiency of error proofing mechanisms [12]. In addition, misincorporation levels can increase due to different stress factors such as nutrient limitation and oxidative stress [13–15]. Multiple proteinogenic amino acid misincorporations have been reported in recombinant proteins produced in mammalian expression systems due to starvation [8, 16, 17]. Wong et al. [15] created a catalogue of these potential amino acid misincorporations in CHO based expression systems including those arising from amino acid starvation. Since amino acid starvation-induced misincorporations can be controlled by replenishing the limiting nutrient, Lin et al. [18] have developed an effective strategy to monitor and address such misincorporations using a combination of amino acid profiles and tandem mass spectrometry.

In addition to proteinogenic amino acids, non-proteinogenic amino acids and rare PTMs can also be detected in the protein sequence [19–21]. One of the more common mammalian non-proteinogenic amino acids is hydroxyproline, a known analog of proline that is found in collagen supporting its triple helix structure and stability [22, 23]. Hydroxyproline is incorporated into collagen by post-translational modification [24]. Hydroxylation of proline occurs after protein synthesis and is catalyzed by the enzyme prolyl hydroxylase [25]. Tyshchuk et al. [21] and Spahr et al. [23] reported the presence of hydroxyproline and a corresponding hydroxylation site in recombinant therapeutics expressed using CHO expression systems. *E*. *coli* prolyl-tRNA synthetase can misincorporate hydroxyproline albeit at much lower rates than proline [26]. To the best of our knowledge, it is unknown if mammalian prolyl-tRNA synthetase can misincorporate hydroxyproline or if the hydroxylated form in expressed recombinant proteins occurs due to post-translational modification alone.

Our work describes the characterization of a subset of proline misincorporations substituted with hydroxyproline, which is found in a recombinant antibody produced in CHO cells using a fed-batch production process. We found hydroxyproline misincorporation in a proline concentration-dependent manner and demonstrated that the mechanism included co-translational misincorporation by using deuterium labelled hydroxyproline. Although misincorporation of non-proteinogenic amino acids is not widely monitored in biotherapeutics, this work suggests that non-proteinogenic amino acids can be misincorporated by mammalian expression systems and the knowledge of the mechanism will be critical to develop effective mitigation strategies.

## Materials and methods

### Cell culture and bioreactor production

Industrially relevant CHO cell lines producing recombinant monoclonal antibody (mAb) were used for all the experiments performed in this study. Cells were cultured in a proprietary basal medium, and scaled up in shake flasks (Corning Inc.) maintained in a 5% CO_2_ incubator at 37°C at 125rpm shaking speed. Bioreactor production was performed using small-scale (Applikon Inc.) and ambr250 (Sartorius AG) bioreactors. A fed-batch production process was carried out by inoculating a production bioreactor with a fixed split ratio from a targeted N-1 culture of appropriate volume. Cells were inoculated in the basal medium and a proprietary feed was added to the bioreactor based on a feeding schedule. Process parameters such as pH, dissolved oxygen, agitation and temperature were controlled. The growth of cells was monitored via viable cell density (VCD) and viability measurements everyday using a Vi-CELL XR automated cell counter (Beckman Coulter). Cell culture supernatants were collected, and the samples were analyzed for titer and metabolite concentrations using a metabolite analyzer (Roche AG). Production was typically carried for 14 days, and final day cell culture samples were purified and submitted for sequence variant analysis.

### Protein A purification

The harvest cell culture fluid samples (HCCF) were purified by affinity chromatography using immobilized Protein A resin (GE Healthcare) according to the manufacturer’s recommended procedure. The mAbs were eluted under acidic conditions with sodium acetate buffer at pH 3.4. The mAb concentration was detected by UV absorption at 280 nm. Protein A purified mAb was used for sequence variant analysis.

### Addition of stable isotope labelled hydroxyproline

Deuterium (3,3,4,5,5 –D5) labelled trans-4-hydroxy-l-proline (Cambridge Isotope Laboratories Inc) at 96% purity was added to the bioreactor feed media after the unlabelled free hydroxyproline was removed from the chemically defined portion of the feed formulation. The concentration of deuterium labelled hydroxyproline was reduced to 85% of the concentration of free hydroxyproline in the original bioreactor feed media. For side-by-side comparisons, the hydroxyproline concentration in the control conditions were adjusted accordingly. This adjustment was performed to ensure that sufficient volume of labelled hydroxyproline feed was available across the duration of the experiment based on limited quantities of the D5 labelled hydroxyproline for two replicate bioreactors. For the labelled hydroxyproline experiment, bioreactors were harvested early on day 8 for misincorporation analysis, as day 8 samples previously demonstrated measurable proline substitutions.

### Spent media amino acid analysis

HCCF samples were analyzed for free amino acids using spent media analysis. Amino acids were derivatized using 6-aminoquinolyl-N-hydroxysuccinimidyl carbamate (AMC), and separated by reversed-phase chromatography using Waters Acquity UPLC BEH C18 column (Waters Corp.) with UV absorbance detection at 260 nm. Individual amino acids in each sample were quantified against each respective standard curve.

### Measurement of labelled and unlabelled hydroxyproline

HCCF samples were diluted using an appropirate diluent and a known concentration of isotopic amino acid standard was added. Separation was performed by capillary electrophoresis using the ZipChip system (908 Devices Inc) with detection by Q Exactive HF-X mass spectrometry. Amino acid concentrations were quantified using the isotopic standard as a single point calibrant.

### LC-MS/MS sequence variant analysis

Protein A purified mAb samples were reduced and alkylated with dithiothreitol (DTT) and iodoacetic acid (IAA), respectively, under denaturing conditions with guanidine hydrochloride followed by digestion using the endoproteinase trypsin. The resulting peptide mixture was separated on a C18 reversed phase column using a gradient of increasing organic concentration in a water/acetonitrile solvent system. Peptides were detected by UV and online mass spectrometry (MS) with a Thermo Q-Exactive mass spectrometer. Peptides were detected in MS survey scans, quantified by extracted ion chromatogram (XIC) integration and validated by MS/MS scans using Thermo Xcalibur software. Sequence variants in these peptides were evaluated in a similar way and the amino acid substitutions were localized using MS/MS scans. The XIC ratio of a sequence variant was calculated as the ratio of the XIC peak area for the variant peptide relative to the total XIC peak area for the variant and nominal peptide. This value per residue was added across the analyzed protein sequence to calculate a summed sequence variant/misincorporation percentage for each substituted amino acid.

## Results

**Fig 1:**
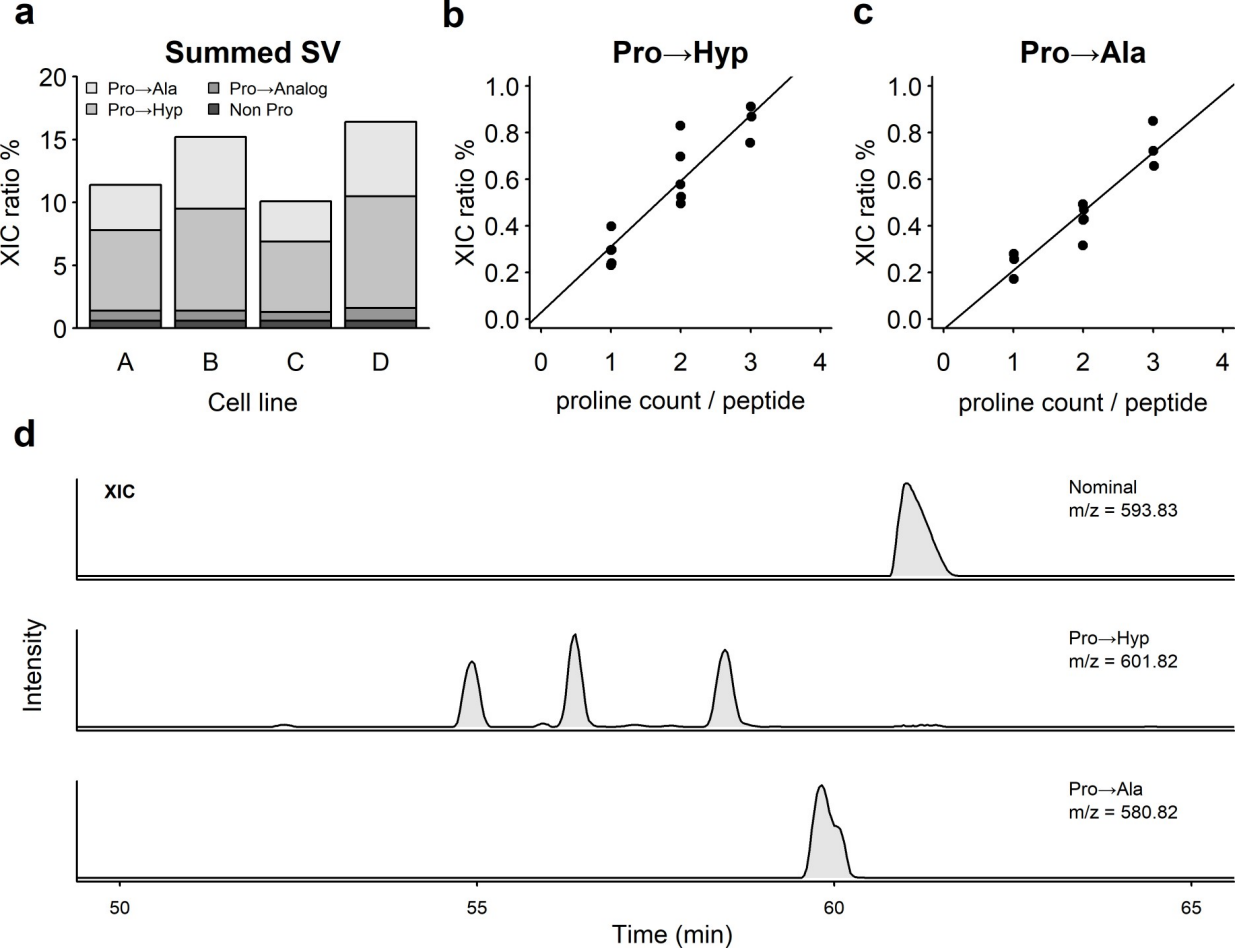
Analysis of proline substitutions observed in recombinant antibodies produced in CHO cell lines. (a) Sum of sequence variant percentages across all proline residues of the recombinant antibody sequence produced from four cell lines A, B, C and D. (b) Hydroxyproline substitution levels were directly proportional to the number of proline residues on peptides analyzed. (c) Alanine substitution levels were directly proportional to the number of proline residues on peptides analyzed. (d) Extracted ion chromatogram (XIC) chromatogram of a peptide showing proline sequence variants with hydroxyproline and alanine replacing proline.

Amino acid substitutions, primarily at proline residues, were observed in recombinant antibodies produced from four CHO cell lines (A, B, C and D) in standard fed-batch production during early-stage development. Fig 1(A) shows the summed extracted ion chromatogram (XIC) ratios of sequence variants observed in protein A purified materials harvested on day 14 of production in bioreactors. Proline was the major amino acid that was substituted across the protein sequence. The predominant substitutions observed were, to alanine and hydroxyproline. Fig 1(B) and 1(C) indicate that the level of summed alanine and hydroxyproline substitutions increase proportionally with the number of proline residues on the peptide. These trends showed that substitutions were distributed across the protein sequence without positional bias for either substituted residue. Representative XIC chromatograms showing distinct peaks for proline, hydroxyproline and alanine are shown in Fig 1(D). MS2 spectra corresponding to the three hydroxyproline peaks are shown in S1 Fig.

**Fig 2:**
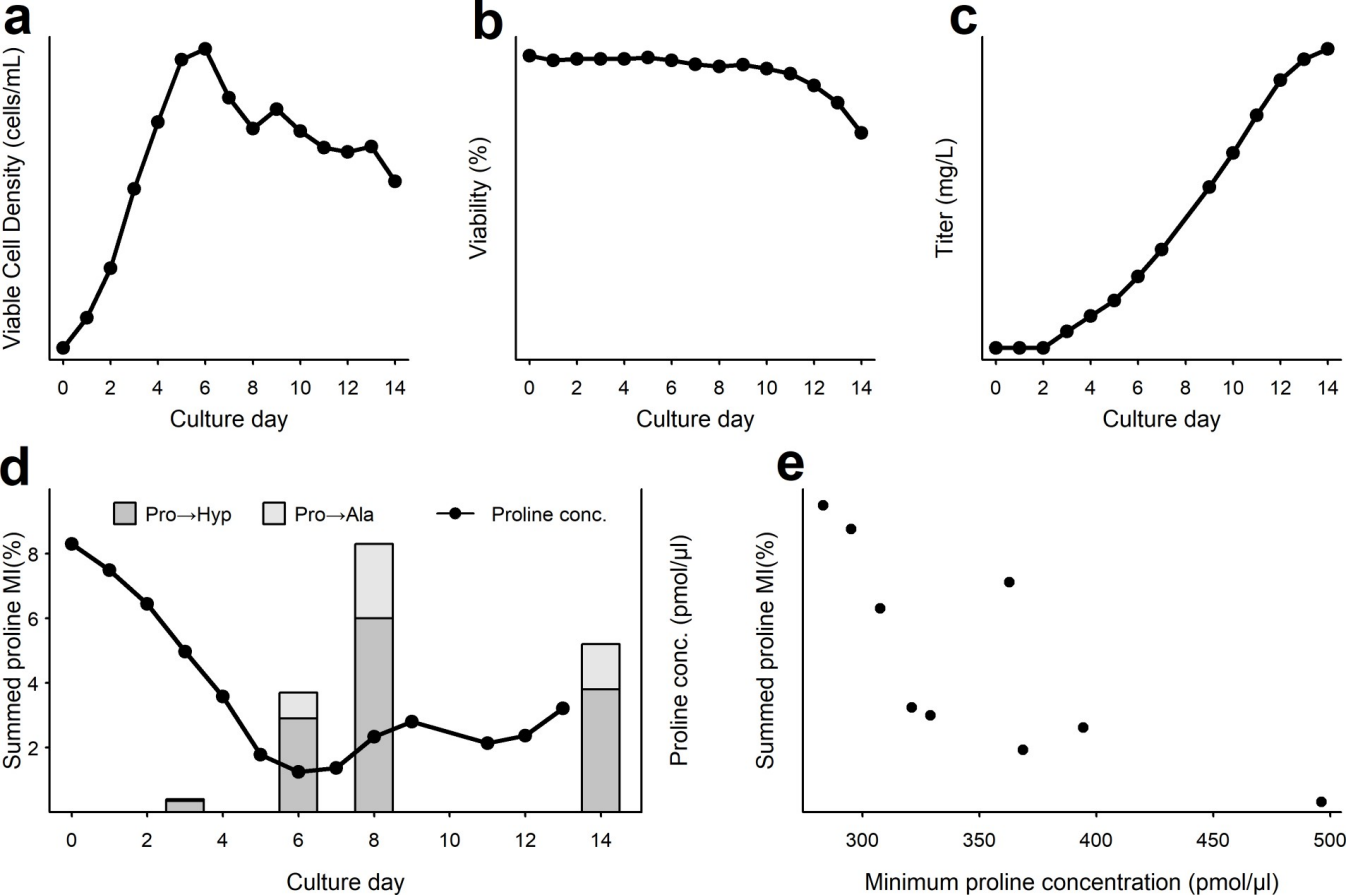
Analysis of cell culture performance and the relationship between proline amino acid levels and substitutions observed for cell line D. Cell culture performance for a single production bioreactor is shown (a) Viable cell density, (b) Viability, (c) Titer. (d) Proline concentration and proline substitutions in the harvest cell culture fluid (HCCF) from the same bioreactor, measured at different time points across culture duration. (e) Relationship between proline substitutions and minimum proline concentration. Multiple bioreactor runs of cell line D were analyzed to derive this relationship.

In order to understand the mechanism of proline substitutions, cell culture performance of cell line D was analyzed further. (Fig 2A–2C) show the production stage viable cell density (VCD), viability and titer profiles for a single bioreactor from a fed batch production experiment. The growth and titer observed were consistent with typical fed-batch production processes. Proline concentrations in harvested cell culture fluid (HCCF) samples were measured everyday using spent media analysis. A minimum concentration of proline was observed for cell line D on day 6, shown in Fig 2(D). We also analyzed intermediate samples collected prior to the final day and observed significant proline substitutions on days 6 and 8 as shown in Fig 2(D). Multiple development runs for cell line D were analyzed to understand the relationship between proline levels and proline substitutions. Summed proline substitutions on day 14 were inversely proportional to the minimum proline concentration in production as shown in Fig 2(E). Higher minimum proline levels in the spent media correlated with lower proline substitutions, regardless of whether the substituted amino acid was alanine or hydroxyproline. The mechanism of proline to alanine misincorporations has been previously described for antibody expressed in CHO cells as a misacylation-based misincorporation [15]. However, hydroxyproline is widely considered to arise from post-translational hydroxylation of proline [24]. Given the random pattern of substitutions and dependency on proline levels for cell line D, we wanted to determine if hydroxyproline could be incorporated directly into recombinant antibody.

**Fig 3:**
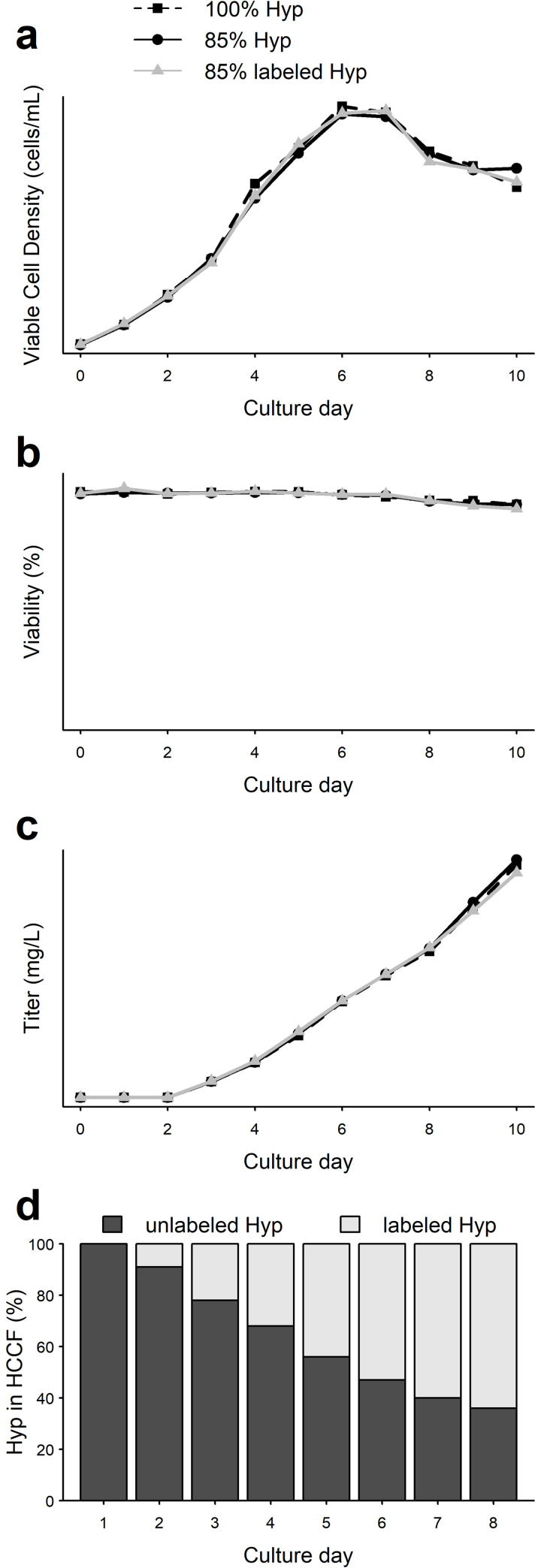
Cell culture performance of bioreactors treated with labeled hydroxyproline compared with other control conditions. (a) Viable cell density (b) Viability (c) Titer (d) Integrated capillary electrophoresis and mass spectrometry analysis of cell culture samples from experimental bioreactors showing unlabeled and labeled hydroxyproline present in the media.

To investigate co-translational misincorporation of hydroxyproline, we replaced hydroxyproline in the cell culture feed medium of cell line D with deuterium labelled hydroxyproline. If the co-translational hypothesis is correct, then we would expect that when proline levels fall below a certain level in cell culture, proline tRNAs will be misacylated with hydroxyproline and hydroxyproline will be misincorporated into the primary sequence of the recombinant protein. The presence of the labelled hydroxyproline can then be verified and quantitated with standard LC-MS/MS methodologies. Due to the labelled hydroxyproline availability, 85% of the original hydroxyproline concentration in the feed was chosen, and a control condition with the same concentration of unlabelled hydroxyproline was included. In addition, a control condition with 100% unlabelled hydroxyproline added to the feed formulation was run to monitor process consistency and ensure comparable performance in this experiment with respect to prior runs. The bioreactors incorporating the isotope-modified feed followed the same feed schedule used previously but with early termination on day 10, and day 8 HCCF samples were used for sequence variant analysis. Cell growth and antibody production in the controls and labelled hydroxyproline bioreactors were consistent as shown in (Fig 3A–3C) demonstrating that the labelled hydroxyproline did not have any significant impact on cell culture performance. Integrated capillary electrophoresis and mass spectrometric analysis of samples from one bioreactor with labelled hydroxyproline feed showed that the ratio of labelled to unlabelled hydroxyproline increased with time with an average of 65% labelled hydroxyproline from day 6 to day 8 as shown in Fig 3(D). This was consistent with increasing amounts of labelled hydroxyproline feed added to the bioreactor.

**Fig 4:**
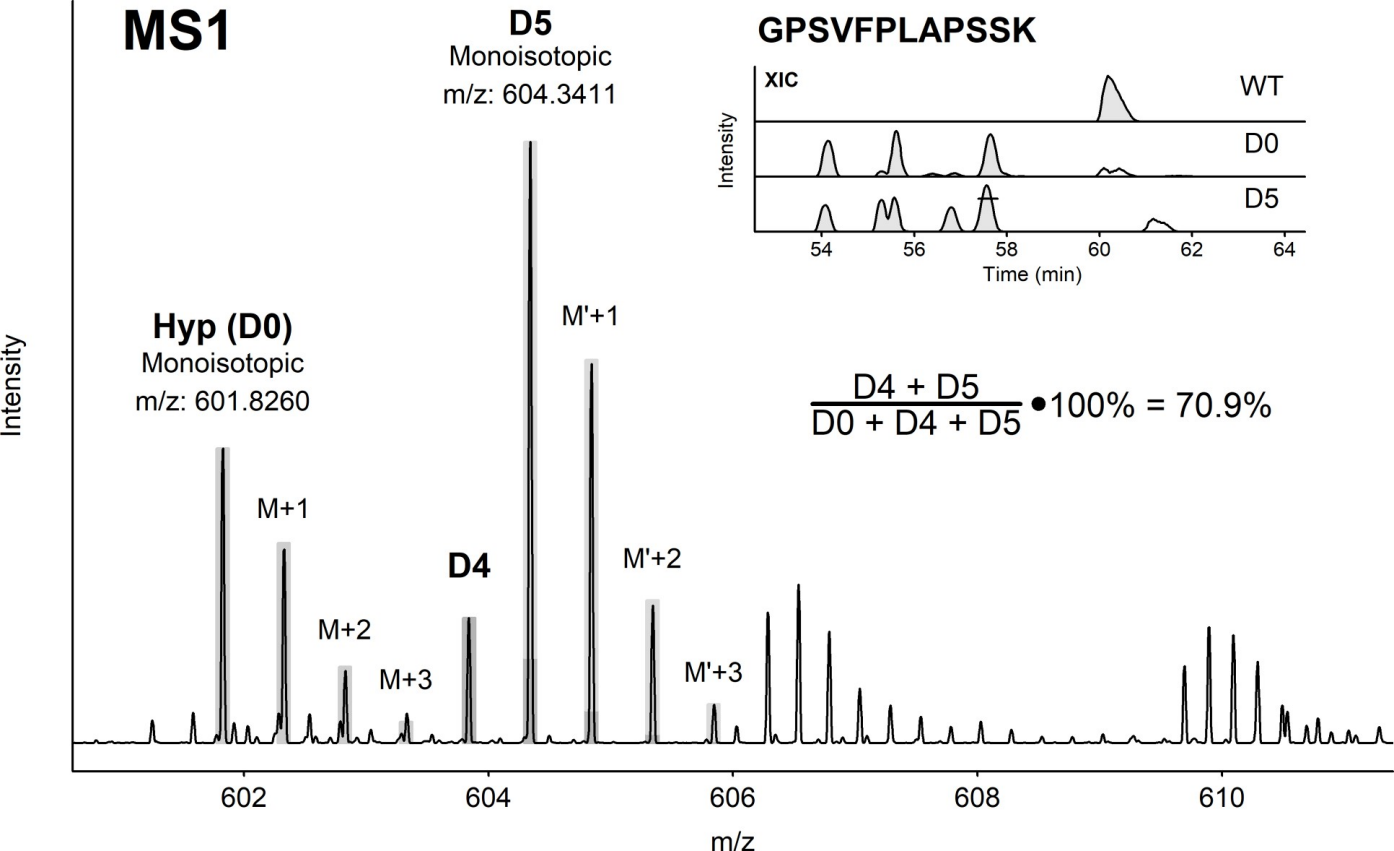
Detection of labeled hydroxyproline in recombinant antibody sequence. Hydroxyproline misincorporation was observed in the antibody as shown by the XIC ratios for a representative peptide (inset). Both unlabeled and labeled hydroxyproline were observed. MS analysis showed the presence of D5 and D4 hydroxyproline.

Sequence variant analysis confirmed proline misincorporation to both alanine and hydroxyproline in all the bioreactors. Misincorporation rates in the 100% control sample were comparable to previous experiments at similar bioreactor scale (11.2% vs 8.5%) and summed proline substitutions was approximately 10% for the 85% hydroxyproline bioreactors. Both unlabelled and labelled hydroxyproline were detected in the HCCF sample from the labelled hydroxyproline fed bioreactor by performing spent media analysis, confirming the misincorporation of hydroxyproline into the recombinant antibody sequence. As expected, based on the purity of the labelled D5-hydroxyproline (96%), a prominent M-1 peak was detected in the MS1 precursor scan which corresponds to the D4-hydroxyproline putative impurity. A binomial model was used to account for under-labelled hydroxyproline and the total hydroxyproline misincorporation level was calculated using the sum of D4- and D5-hydroxyproline intensity (Fig 4). MS/MS spectra for D0 and D5-hydroxyproline are shown in S2 Fig. Unlabelled hydroxyproline substitutions were also detected in the labelled sample consistent with the presence of unlabelled hydroxyproline in the bioreactor cell culture media. The portion of labelled hydroxyproline was stable across the primary sequence as shown in Table 1. This data established that hydroxyproline can be co-translationally misincorporated into proteins produced in mammalian expression systems.

**Table 1:** Percentage of labeled hydroxyproline misincorporations observed in a set of random peptides chosen from the recombinant antibody sequence.

Peptide sequence	number of proline residues	labeled hyp bioreactor 1	labeled hyp bioreactor 2
VEAEDVGVYYCSQSTHVPPTFGQGTK	2	68.7	65.7
TVAAPSVFIFPPSDEQLK	3	69.4	68.8
VYACEVTHQGLSSPVTK	1	70.7	71
GPSVFPLAPSSK	2	70.9	71.1
THTCPPCPAPELLGGPSVFLFPPKPK	8	69.8	69.2
TPEVTCVVVDVSHEDPEVK	2	70.8	69.6
ALPAPIEK	2	70.5	71.4
GFYPSDIAVEWESNGQPENNYK	2	72.4	72.9
TTPPVLDSDGSFFLYSK	2	68.6	68.6

## Discussion

In this study, we identified proline sequence variants substituted with hydroxyproline in recombinant antibodies produced in CHO cells and investigated the mechanism of misincorporation. Sequence variants mainly arise from DNA mutations, post-translational modifications or mistranslation due to tRNA misacylation or misdecoding (codon mispairing or wobble) [27]. Misincorporations based on misacylation or misdecoding reflect a fundamental limit of the fidelity in the protein synthesis machinery resulting in sequence variants that are not specific to a particular residue and are distributed across the protein sequence unlike DNA mutations. Sequence variants caused by codon mispairing occur based on codon usage in the sequence [7, 28].These sequence variants are therefore not altered based on the levels of amino acid in the cell supernatant. The proline substitutions observed in this study were randomly distributed across the protein sequence and the total levels correlated with the amount of free proline in the cell culture supernatant. The pattern and variation in summed sequence variants observed in this study suggested that these were misincorporations caused by amino acid starvation.

Sequence variants caused by misincorporation of non-canonical amino acids like norvaline and norleucine have been previously reported in recombinant proteins produced in *E*. *coli* [29]. In addition to norvaline and norleucine, proline misincorporation has been demonstrated with non-proteinogenic amino acids. Hydroxyproline and azetidine are mischarged by the prolyl tRNA and misincorporated into recombinant proteins expressed in *E*. *coli* [26]. Non-proteinogenic amino acid misincorporation has been observed in mammalian systems as well. Beta-methylamino-l-alanine (BMAA) and azetidine-2-carboxylic acid are among several plant based non-canonical amino acids that can be incorporated into endogenous proteins in humans through the food chain [30–33]. Recombinant proteins expressed in rabbit reticulocytes and human (HEK) cells can also misincorporate the proline analog azetidine-2-carboxylic acid and, interestingly, the human prolyl-tRNA synthetase appears to lack proof-reading activity required to reject non-proteinogenic amino acids [34–36].

Non-proteinogenic amino acid misincorporation requires sufficient concentration of the substituting amino acid in comparison with the cognate amino acid, as well as the propensity for mischarging by amino acyl-tRNA synthetase. Misincorporation of non-proteinogenic amino acids in recombinant proteins produced in *E*. *coli* can be driven by the lack of cognate amino acid as well as factors that promote synthesis of the non-cognate amino acids in the culture medium [37–40]. Spent media analysis of the fed batch bioreactor HCCF for cell line D (growth from this experiment reported in Fig 2), confirmed that hydroxyproline is present at a of 1295 picomoles per microliter showing this could be a source for misincorporation. Cell growth media developed for mammalian cells has been shown to include hydroxyproline in the formulation [41, 42]. Additionally, bioreactor feed formulations can include hydrolysates derived from plant sources such as soybean which is a rich source of hydroxyproline [43–45].

Given the presence of hydroxyproline and prior evidence of prolyl tRNA mischarging proline analogs, we tested the hypothesis that the hydroxyproline substitutions were co-translational misincorporations. Our data showed that hydroxyproline can be incorporated into CHO expressed antibody, most likely via co-translational misincorporation. Unlabelled hydroxyproline was also found in the recombinant protein sequence consistent with the presence of unlabelled hydroxyproline in cell culture media and feed. The fraction of labelled hydroxyproline relative to total hydroxyproline in HCCF was measured using spent media analysis, at an average of 59 percent from days 6 to 8. Analysis of multiple peptides showed that the fraction of labelled hydroxyproline relative to total hydroxyproline was at 65.7–72.9 percent suggesting that a significant fraction of unlabelled hydroxyproline was present in the protein sequence similar to the percentage measured in the HCCF using spent media analysis. The low level of misincorporations distributed randomly across the protein sequence, dependence on proline levels and the incorporation of labelled hydroxyproline suggests co-translational misincorporation as a mechanism for hydroxyproline incorporation into recombinant proteins produced in CHO cells.

This study has conclusively shown that CHO cells, the most widely used expression host for recombinant therapeutic expression, can incorporate non-proteinogenic amino acid hydroxyproline into proteins using an additional mechanism, which is mistranslation based misincorporation. This finding is significant since non-proteinogenic amino acid misincorporation is not routinely monitored during analytical characterization, and cell culture components can potentially provide a source for some of these amino acids, like hydroxyproline. This route of misincorporation into recombinant proteins necessitates a different approach to the development of mitigation and control strategies, for example, controlling or eliminating the levels of non-cognate amino acids available in the cell culture media. Mitigating co-translational misincorporation of non-proteinogenic amino acids will require development of sensitive detection assays to confirm the mechanism and analyze the relationship between amino acid concentrations and misincorporation levels. Understanding the risks associated with the incidence and impact of these types of misincorporation will be necessary to develop a sound control strategy for controlling misincorporation in recombinant protein-based therapeutics.

## Supporting information

S1 Fig Representative MS2 spectra in support of hyp localization for peptide GPSVFPLAPSSK. An MS2 spectra corresponding to each of the peaks from Fig 1D are shown. Prominent b and y-ions are annotated and the ion providing hyp localization is circled.
(TIF)

S2 Fig Representative MS2 spectra in support of unlabeled (D0) and labeled (D5) hyp identification. MS2 spectra corresponding to the D0 and D5 species taken at the same retention time are shown with b and y-ion annotation. The offset caused by the labeling is visible in the b2 ion.
(TIF)
