# Supplementary figures and images for: Evidence for co-translational misincorporation of non-canonical amino acid hydroxyproline in recombinant antibodies produced in Chinese Hamster Ovary (CHO) cell lines
Source: PLoS One. 2020 Oct 29;15(10):e0241250. doi: 10.1371/journal.pone.0241250 (PMC7595273; doi:10.1371/journal.pone.0241250)

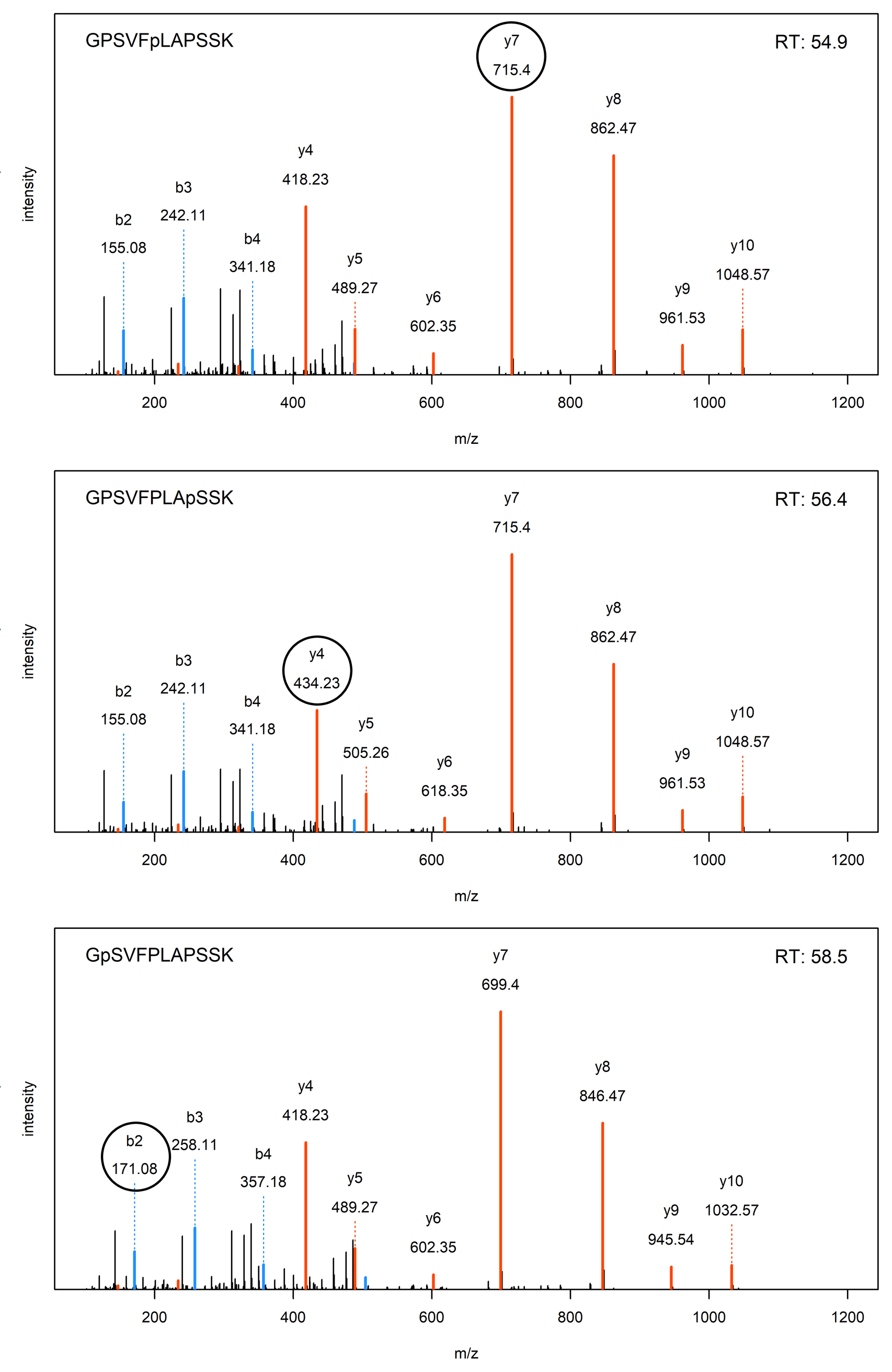

Supplement: S1 Fig — An MS2 spectra corresponding to each of the peaks from Fig 1D are shown. Prominent b and y-ions are annotated and the ion providing hyp localization is circled. (TIF) [file pone.0241250.s001.tif]

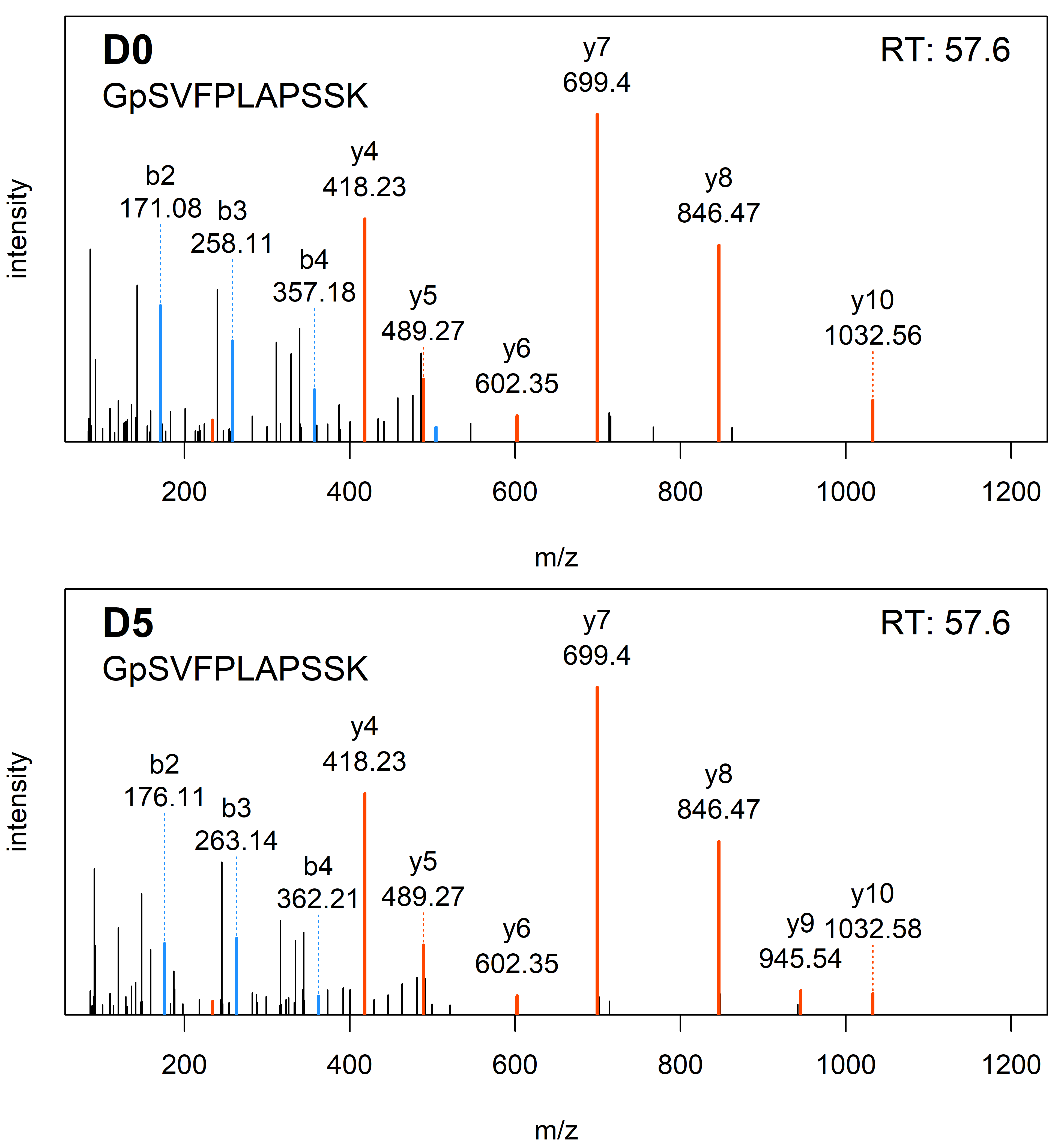

Supplement: S2 Fig — MS2 spectra corresponding to the D0 and D5 species taken at the same retention time are shown with b and y-ion annotation. The offset caused by the labeling is visible in the b2 ion. (TIF) [file pone.0241250.s002.tif]
